# Robust Dual-Stream Diagnosis Network for Ultrasound Breast Tumor Classification with Cross-Domain Segmentation Priors

**DOI:** 10.3390/s26030974

**Published:** 2026-02-02

**Authors:** Xiaokai Jiang, Xuewen Ding, Jinying Ma, Chunyu Liu, Xinyi Li

**Affiliations:** School of Electronic Engineering, Tianjin University of Technology and Education, Tianjin 300222, China; 0421231024@tute.edu.cn (X.J.); majinying@tute.edu.cn (J.M.); 0421231009@tute.edu.cn (C.L.); 0422231045@tute.edu.cn (X.L.)

**Keywords:** ultrasound image analysis, breast cancer diagnosis, segmentation prior guidance, feature fusion, transformer block

## Abstract

Ultrasound imaging is widely used for early breast cancer screening to enhance patient survival. However, interpreting these images is inherently challenging due to speckle noise, low lesion-to-tissue contrast, and highly variable tumor morphology within complex anatomical structures. Additionally, variations in image characteristics across institutions and devices further impede the development of robust and generalizable computer-aided diagnostic systems. To alleviate these issues, this paper presents a cross-domain segmentation prior guided classification strategy for robust breast tumor diagnosis in ultrasound imaging, implemented through a novel Dual-Stream Diagnosis Network (DSDNet). DSDNet adopts a decoupled dual-stream architecture, where a frozen segmentation branch supplies spatial priors to guide the classification backbone. This design enables stable and accurate performance across diverse imaging conditions and clinical settings. To realize the proposed DSDNet framework, three novel modules are created. The Dual-Stream Mask Attention (DSMA) module enhances lesion priors by jointly modeling foreground and background cues. The Segmentation Prior Guidance Fusion (SPGF) module integrates multi-scale priors into the classification backbone using cross-domain spatial cues, improving tumor morphology representation. The Mamba-Inspired Linear Transformer (MILT) block, built upon the Mamba-Inspired Linear Attention (MILA) mechanism, serves as an efficient attention-based feature extractor. On the BUSI, BUS, and GDPH_SYSUCC datasets, DSDNet achieves ACC values of 0.878, 0.836, and 0.882, and Recall scores of 0.866, 0.789, and 0.878, respectively. These results highlight the effectiveness and strong classification performance of our method in ultrasound breast cancer diagnosis.

## 1. Introduction

Breast cancer represents the most frequently diagnosed malignancy among women and ranks among the top causes of cancer mortality [1]. In 2022, it constituted 11.6 percent of all new cancer cases globally and was responsible for 6.9 percent of cancer-related deaths worldwide [2]. Early detection and accurate diagnosis are paramount for reducing mortality and improving survival, as they enable prompt intervention and the customization of therapeutic plans [3,4]. Among the various imaging modalities employed in early breast tumor diagnosis, including Magnetic Resonance Imaging (MRI), Computed Tomography (CT), ultrasound imaging, and digital mammography, ultrasound imaging stands out for its cost efficiency, real-time imaging capability, high sensitivity, and absence of ionizing radiation [5,6]. Interpreting breast ultrasound images poses several intrinsic challenges. These images are often degraded by speckle noise and low contrast between lesion and surrounding tissue, while tumor morphology can vary widely and be embedded within complex anatomical structures [7]. As a result, reliable diagnosis typically requires the skill and experience of well-trained radiologists to discern subtle textural differences and accurately delineate lesion boundaries. Therefore, it is essential to develop robust and efficient Computer-Aided Diagnostic (CAD) algorithms to assist radiologists in interpreting breast ultrasound scans.

Traditional CAD systems for breast ultrasound predominantly rely on handcrafted feature extraction followed by classical machine learning classifiers. Traditional computer-aided diagnosis of breast ultrasound typically begins with the manual design and extraction of features that characterize lesion morphology, texture patterns, intensity statistics and other tumor-specific attributes. This labor-intensive process relies heavily on domain expertise. Classical image processing techniques, such as intensity thresholding [8], watershed segmentation [9], region growing [10] and edge detection [11], are then employed to delineate tumor and normal tissue regions. For classification, these handcrafted feature vectors serve as inputs to conventional machine learning algorithms. Methods such as decision trees [12], support vector machines [13], random forests [14] and k-nearest neighbors [15] are trained to distinguish benign from malignant breast lesions based on the extracted descriptors. While traditional CAD systems demand minimal computation and data, they rely on handcrafted feature sets that require extensive expert input and often fail to adapt to varying image quality or complex anatomy. Consequently, these methods exhibit poor generalization, limited sensitivity to subtle lesion characteristics, and overall suboptimal diagnostic accuracy.

Deep learning has demonstrated remarkable effectiveness across diverse domains, as evidenced by its ability to enable efficient feature representation in multi-agent systems [16], enhance decision-making through attention mechanisms [17], and improve multi-modal data integration for complex prediction tasks [18]. Motivated by the limitations of handcrafted approaches and the rapid advances in deep learning, recent research has turned to deep learning for ultrasound breast tumor classification, leveraging its ability to automatically learn hierarchical features and deliver superior accuracy compared with traditional CAD methods. Representative architectures span from convolutional networks such as VGG [19], ResNet [20] and Inception variants [21,22] to transformer-based models like ViT [23]. For instance, Sirjani et al. [24] redesigned the InceptionV3 architecture for ultrasound breast tumor classification by tuning the number of Inception modules. This tailored network demonstrated superior classification accuracy on ultrasound images compared to the standard InceptionV3 model. Deb et al. [25] proposed a rank-based ensemble framework that integrates pretrained VGG, DenseNet, Inception, and Xception networks. Their method employs tanh, sigmoid, and exponential functions to fuse individual model outputs, and it outperforms conventional ensemble strategies in breast ultrasound image classification. Alhussan et al. [26] devised a hybrid framework that employs metaheuristic optimization for feature selection in tandem with deep learning models, leading to more robust feature sets prior to ultrasound image classification. Chen et al. [27] introduced a technique to mitigate classification-layer dependencies by enforcing semantic consistency in the learned feature space, improving the model’s ability to generalize across diverse datasets, including breast ultrasound images. As Transformer architectures mature and achieve notable success in computer vision, their adoption in ultrasound breast tumor classification has become increasingly widespread. For example, Manzari et al. [28] adapted proven natural language processing strategies to design a CNN-Transformer hybrid that achieves both high robustness and computational efficiency in the classification of ultrasound images. Gheflati et al. [29] explored the application of a pretrained Vision Transformer to breast ultrasound classification. They enhanced model performance through extensive data augmentation and addressed inherent class imbalance by employing a weighted cross-entropy loss. Üzen et al. [30] present SW-ForkNet, a hybrid model that extends DenseNet121 by introducing parallel spatial and long-context branches. The spatial branch incorporates a Squeeze-and-Excitation module to retain fine-grained details, while the long-context branch utilizes a Swin Transformer to capture global dependencies. The extracted features are fused through element-wise addition, enabling efficient and accurate breast tumor classification. Recent advances in ultrasound breast tumor classification have introduced tailored network architectures, ensemble learning strategies, optimized feature engineering, and Transformer-based attention mechanisms. These developments have significantly improved the robustness, generalization, and accuracy of diagnostic models. However, many of these methods tend to overlook the critical role of lesion segmentation in the diagnostic process. Without incorporating segmentation information, models often lack the ability to perceive tumor morphology as a whole. This limitation reduces their effectiveness in identifying low-contrast boundaries and capturing the complex anatomical structures commonly found in breast ultrasound images.

To address this limitation, several recent models have sought to integrate segmentation and classification tasks, leveraging features derived from the lesion segmentation process or the segmentation results themselves to guide the classification process. For instance, Zhou et al. [31] developed a unified architecture designed to simultaneously perform classification and segmentation on volumetric breast ultrasound data. By incorporating an iterative refinement strategy during training, their method effectively reduced boundary ambiguity and noise, resulting in performance improvements across both tasks when compared to models trained for classification or segmentation alone. Mishra et al. [32] introduced a method that leverages feature maps from both the encoder and decoder stages of a segmentation network to support not only accurate tumor boundary delineation but also effective tumor classification, thereby enabling joint optimization of both tasks. Kang et al. [33] developed a multi-stage, multi-task learning approach designed to improve both segmentation and classification of thyroid nodules in ultrasound images by enforcing consistency within and between the tasks during training. Zhu et al. [34] propose a novel multi-task learning framework, DBL-Net, to simultaneously perform breast tumor segmentation and classification in ultrasound images. They integrate the frequency domain information into the analysis pipeline. Aumente-Maestro et al. [35] propose an end-to-end multi-task learning framework that jointly performs breast lesion segmentation and classification in ultrasound images, addressing key limitations in existing deep learning systems such as data bias, exclusion of non-tumor cases, and single-task constraints. Jiang et al. [36] propose a novel deep learning-based multi-stage diagnostic framework that leverages segmentation knowledge to enhance tumor classification in ultrasound images, particularly for breast and thyroid cancers. Crucially, the refined segmentation results are integrated into the classification stage to enrich multimodal feature extraction, thereby improving diagnostic accuracy. While these multi-task methods acknowledge the importance of lesion segmentation in the diagnostic process of breast tumors, they typically rely on datasets that contain both pixel-level segmentation masks and image-level classification labels for the same samples. However, such fully annotated datasets are costly to obtain and remain limited in availability. Moreover, due to significant variations in grayscale distribution, resolution, and probe angle across ultrasound images acquired from different institutions and devices, these models are prone to overfitting to domain-specific features. As a result, their performance may fluctuate drastically when deployed in unseen clinical settings, limiting their generalizability in cross-domain scenarios.

To tackle these challenges, a dedicated segmentation branch is first pretrained on an external ultrasound breast tumor segmentation dataset to obtain robust, cross-domain morphology priors. This frozen branch is then integrated into the classification backbone to form the Dual-Stream Diagnosis Network (DSDNet). During classification training, the segmentation branch supplies multi-scale spatial priors that guide the backbone toward capturing the global tumor morphology, thereby leveraging lesion masks to enhance diagnostic accuracy. By keeping the segmentation parameters frozen, the spatial priors remain unaffected by classification gradients, ensuring stable and reliable guidance. Because the segmentation branch is pretrained on cross-domain data, it is less prone to overfitting to a single domain and exhibits more robust performance when applied to data from new centers. Furthermore, to enhance diagnostic accuracy, novel modules are created at the interface between the segmentation and classification streams to more effectively leverage the segmentation priors. A new feature-extraction component is integrated into the classification backbone, augmenting the ability of DSDNet to delineate low-contrast tumor boundaries and capture complex, variable anatomical structures in breast ultrasound images.

The main contributions of this work can be summarized as follows:

1. We propose a cross-domain segmentation prior guided classification strategy and develop a DSDNet, in which a frozen segmentation branch provides robust spatial priors to guide classification. This decoupled dual-stream design effectively enhances classification robustness across heterogeneous ultrasound imaging conditions, including different devices and clinical centers.
2. To fully exploit segmentation priors, we design two dedicated modules: the Dual-Stream Mask Attention (DSMA) module, which jointly models foreground–background dependencies to enhance lesion-aware representations, and the Segmentation Prior Guidance Fusion (SPGF) module, which injects multi-scale segmentation priors into the classification backbone. Together, they enable more accurate and stable tumor representation learning.
3. We introduce a Mamba-Inspired Linear Attention (MILA) mechanism and build a Mamba-Inspired Linear Transformer (MILT) block as the core of the classification branch. MILA integrates the advantages of Mamba into a linear attention framework, enabling efficient modeling of long-range dependencies and fine-grained tumor structures in breast ultrasound images.

This paper is organized as follows. Section 1 reviews the clinical importance of ultrasound breast tumor classification and the limitations of current approaches. Section 2 introduces the DSDNet, providing an overview of its architecture and core modules. Section 3 details the experimental design, including the datasets, implementation settings, and evaluation metrics. Section 4 presents and analyzes quantitative and qualitative results, comparing DSDNet with state-of-the-art methods. Finally, Section 5 offers concluding remarks and suggests avenues for future research.

## 2. Methodology

### 2.1. Overall Network Architecture

Breast ultrasound images present several challenging characteristics, including low contrast, severe speckle noise, blurred lesion boundaries, and significant appearance variations caused by different imaging devices and clinical centers. Among these issues, inaccurate lesion localization and poor cross-domain generalization are the key problems that limit reliable tumor classification. To address these challenges, our method explicitly leverages segmentation priors to provide stable spatial guidance for classification.

**Figure 1:**
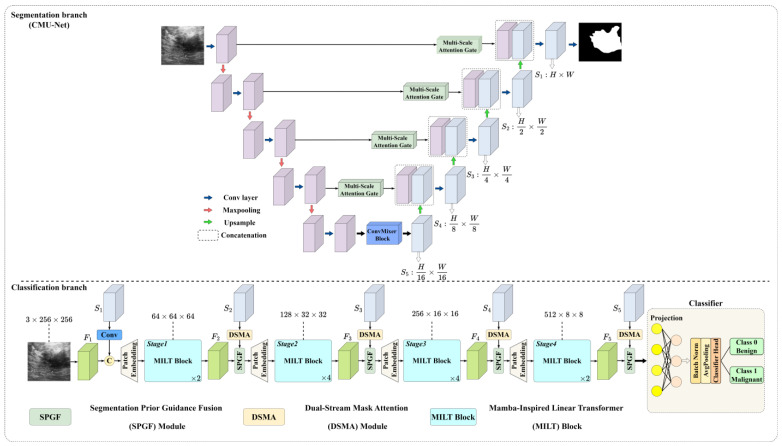
The overall network architecture of the proposed Dual-Stream Diagnosis Network (DSDNet).

The overall architecture of DSDNet, as shown in Figure 1, consists of two decoupled but synergistic streams: a frozen, pretrained segmentation branch and a parallel, trainable classification branch. These streams communicate exclusively through three specialized modules that inject and utilize segmentation priors.

First, the segmentation stream is trained on an external breast ultrasound segmentation dataset to learn robust, multi-scale lesion priors; these priors are then frozen and employed throughout classification training to provide stable spatial guidance. At each of the four resolution levels in the classification backbone, the segmentation priors are first refined by the DSMA module, which employs separate attention branches for the mask and its surrounding context. By fusing foreground and background information, DSMA sharpens the focus on diagnostically relevant tumor regions and attenuates distracting background textures, thereby aligning the segmentation guidance more closely with the feature extraction of the classification backbone. The refined priors are then merged with the classification feature maps by the SPGF module, which uses cross-domain segmentation priors supplied by the segmentation branch to guide the network toward a more comprehensive and accurate representation of breast tumor morphology in ultrasound images. Finally, each stage concludes with a MILT block that leverages the MILA mechanism, which is inspired by the Mamba architecture and embeds its core strengths within an efficient linear attention framework. By adopting a refined block design and replacing the conventional forget gate with a parallelizable positional encoding scheme, the MILT block markedly improves the extraction of fine tumor boundaries and the modeling of complex, broadly distributed anatomical structures in breast ultrasound images. In the classification backbone, hierarchical feature representations (denoted as F1–F5 in Figure 1) are progressively extracted through successive stages, where each stage increases semantic abstraction while retaining spatial information.

After several guided stages, each of which increases semantic depth while preserving spatial precision, the resulting high-level feature (F5) is projected into a classifier to yield benign versus malignant predictions. By decoupling the frozen segmentation priors from the classification gradients and weaving them through a structured, dual-stream pipeline, DSDNet attains robust, high-fidelity tumor delineation and stable performance across heterogeneous ultrasound data sources.

### 2.2. Dual-Stream Mask Attention Module

To effectively harness the spatial priors provided by the frozen segmentation branch, the DSMA module is inserted before each fusion stage of the classification backbone. The DSMA module operates on the raw segmentation priors, processing them through parallel attention pathways that learn to balance foreground and background information. By computing complementary attention maps for the mask and its surrounding feature context, DSMA refines the segmentation prior representations before they are fused into the backbone, ensuring that salient tumor cues are amplified while complex background structures are suppressed. The following section details the internal architecture and computational flow of the DSMA module.

**Figure 2:**
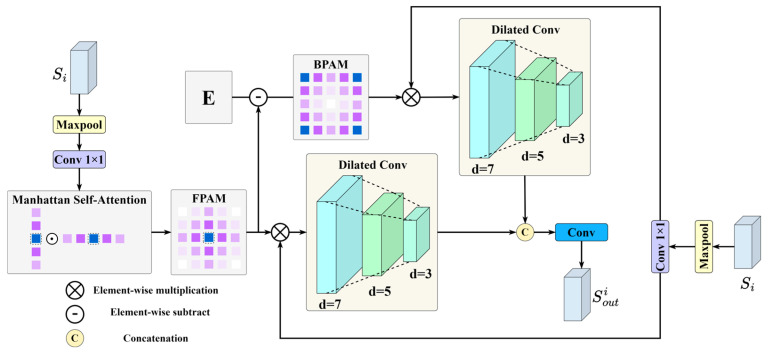
The schematic of the Dual-Stream Mask Attention (DSMA) module.

As illustrated in Figure 2, the DSMA module begins by encoding fine-grained spatial relationships within the segmentation prior $S_{i}$ using Manhattan Self-Attention (MaSA) [37], which extends the standard self-attention mechanism by integrating a distance decay term into the attention weights. The distance matrix is reformulated as follows:

(1) $$D_{nm}^{2d}=\gamma^{\left|x_{n}-x_{m}\right|+\left|y_{n}-y_{m}\right|},$$

where $D^{2d}$ is the two-dimensional bidirectional distance matrix. Thus, the MaSA computation is formulated as follows:

(2) $$MaSA\left(X\right)=\left(\mathrm{Softmax}\left(QK^{T}\right)⊙D^{2d}\right)V,$$

where ⊙ denotes element-wise multiplication. This scheme preserves global context while biasing attention toward spatially proximate tokens, thereby enhancing both local detail and long-range dependencies. The DSMA module employs MaSA to generate a Foreground Priority Attention Map (FPAM), which emphasizes salient tumor regions in the segmentation prior. The FPAM is subtracted from an all-one matrix E to derive the Background Priority Attention Map (BPAM), thereby enabling the modeling of both foreground and background cues. This process is computed as follows:

(3) $$\begin{array}{l} A^{F}=MaSA\left(Conv\left(Maxpool\left(S_{i}\right)\right)\right),\\ A^{B}=E-A^{F}, \end{array}$$

where $S_{i}$ denotes the input segmentation prior, $A^{F}$ denotes the FPAM, $A^{B}$ represents the BPAM, $Maxpool\left(\cdot \right)$ refers to the max pooling operation, $Conv\left(\cdot \right)$ corresponds to the 1 × 1 convolution operation, and E is a matrix of ones with the same spatial dimensions as the FPAM. The FPAM and BPAM are then fused with the segmentation prior $S_{i}$ through element-wise multiplication, integrating fine-grained boundary information and yielding a more discriminative feature representation. To further enhance sensitivity to complex lesion regions within the segmentation prior, a multi-scale convolutional block is applied, capturing both detailed local structures and the broader tumor context. Finally, the foreground and background features are concatenated and passed through a convolutional layer to generate the output of the DSMA module. The process is formulated as follows:

(4) $$\begin{array}{l} S_{i}^{\prime }=D\left(A^{F}⊙Conv\left(Maxpool\left(S_{i}\right)\right)\right),\\ S_{i}^{\prime \prime }=D\left(A^{B}⊙Conv\left(Maxpool\left(S_{i}\right)\right)\right),\\ S_{out}^{i}=Conv\left(Cat\left(S_{i}^{\prime },S_{i}^{\prime \prime }\right)\right), \end{array}$$

where $D\left(\cdot \right)$ denotes a set of dilated convolutions with dilation rates of 3, 5, and 7, $Cat\left(\cdot \right)$ represents the concatenation operation, and $S_{out}^{i}$ refers to the output of the DSMA module.

### 2.3. Segmentation Prior Guidance Fusion Module

To effectively leverage the pretrained segmentation branch within the classification pipeline, we propose the SPGF module. The SPGF module is inserted at multiple depths in the classification backbone, where it receives two inputs: an intermediate feature map from the classifier and a corresponding scale segmentation prior from the frozen segmentation branch. By adaptively merging these inputs through a segmentation prior self-attention mechanism, the SPGF module directs the representational focus of the backbone to actual lesion regions and concurrently attenuates irrelevant background activations. In the following, we detail the internal architecture of the SPGF module and describe its fusion strategy.

**Figure 3:**
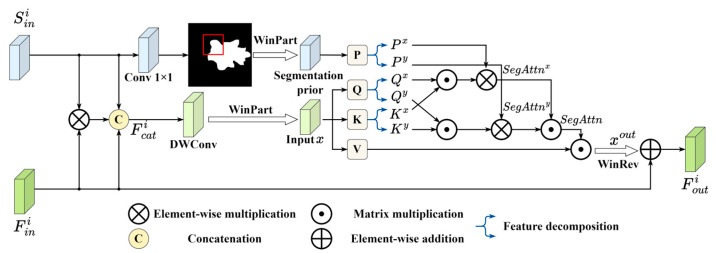
The schematic of the Segmentation Prior Guidance Fusion (SPGF) module.

First, as shown in Figure 3, the segmentation prior feature map is combined with the corresponding classification feature map via element-wise multiplication. The resulting product is then concatenated with the original classification feature map along the channel dimension. The output $F_{cat}^{i}$ is then passed through a depth-wise convolution followed by the Window Partition (WinPart) operation to produce one input x for the Segmentation Prior Self-Attention (SPSA). In parallel, the raw segmentation prior feature map $S_{in}^{i}$ is processed by the convolution to generate the segmentation mask, which is then processed by WinPart to yield the second SPSA input segmentation prior P. The input x is first linearly projected to obtain the query, key, and value matrices Q, K, and V. To facilitate the SPSA computation, Q, K and the segmentation prior P are decomposed along the horizontal axis into components $Q^{x}$, $K^{x}$, and $P^{x}$, and along the vertical axis into components $Q^{y}$, $K^{y}$, and $P^{y}$. Next, the segmentation prior is used to modulate attention in each direction, producing the horizontal attention map $SegAttn^{x}$ and the vertical attention map $SegAttn^{y}$. Finally, these two directional attention maps are fused and applied to V, yielding the SPSA output $x^{out}$. Formally, the procedure can be written as:

(5) $$\begin{array}{l} SegAttn^{x}=softmax\left(Q^{x}{\left(K^{x}\right)}^{T}\right)⊙P^{x},\\ SegAttn^{y}=softmax\left(Q^{y}{\left(K^{y}\right)}^{T}\right)⊙P^{y},\\ x^{out}=SegAttn^{x}{\left(SegAttn^{y}V\right)}^{T}, \end{array}$$

where the $softmax\left(\cdot \right)$ represents the computation of normalized attention weights, and ⊙ denotes element-wise multiplication. Finally, the SPSA output is restored to the original spatial layout via Window Reverse (WinRev) and merged with the classification feature $F_{in}^{i}$ through a residual connection to produce the SPGF module output $F_{out}^{i}$.

### 2.4. Mamba-Inspired Linear Transformer Block

**Figure 4:**
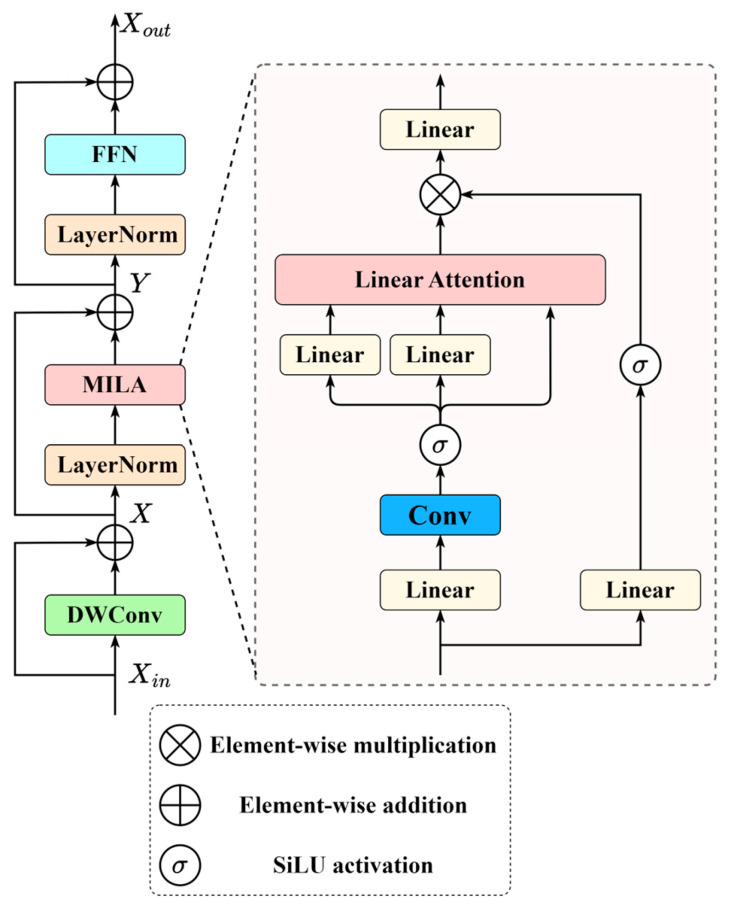
The schematic of the Mamba-Inspired Linear Transformer (MILT) block.

To enable DSDNet to better focus on tumor boundaries in ultrasound images and efficiently model the scattered, non-structured global contextual information in breast ultrasound images while maintaining linear complexity and parallelizable computation, this paper introduces the MILA mechanism. Built upon MILA, the MILT block is proposed as a core component. As illustrated in Figure 4, the MILT block consists of a Depth-Wise Convolution (DWConv), Layer Normalization (LN), the MILA module, a Feed-Forward Network (FFN), and residual connections.

The MILA inherits the efficiency of linear attention while drawing inspiration from the Mamba architecture, particularly its forget-gate mechanism and structured block design. This allows effective extraction of complex anatomical structures and low-contrast tumor boundaries commonly observed in breast ultrasound imaging. In the standard Transformer framework, self-attention is defined as:

(6) $$Attention\left(Q,K,V\right)=softmax\left(\frac{QK^{T}}{\sqrt{d}}\right)V,$$

where $Q,K,V\in {\mathbb{R}}^{N\times d}$ are the query, key, and value matrices, and N is the sequence length. This mechanism captures global dependencies effectively but suffers from quadratic complexity $O\left(N^{2}\right)$, making it computationally expensive for long sequences or high-resolution medical images. To address this issue, linear attention approximates the softmax operation using a kernel-based feature mapping $\phi \left(\cdot \right)$, enabling the reformulated computation:

(7) $$Linear\;Attention\left(Q,K,V\right)=\phi \left(Q\right)\phi {\left(K\right)}^{T}V=\phi \left(Q\right)\left(\sum_{j=1}^{N}\phi {\left(K_{j}\right)}^{T}V_{j}\right).$$

The term inside the parentheses is independent of the query index i and depends solely on the set of key-value pairs ${\left\{\left(\phi \left(K_{j}\right),V_{j}\right)\right\}}_{j=1}^{N}$. Consequently, linear attention defines two precomputed accumulators:

(8) $$\begin{array}{l} S=\sum_{j=1}^{N}\phi {\left(K_{j}\right)}^{T}V_{j},\\ Z=\sum_{j=1}^{N}\phi {\left(K_{j}\right)}^{T}, \end{array}$$

which aggregate all key-value contributions and all key contributions, respectively. For each query position i, linear attention then computes its output as:

(9) $$y_{i}=\frac{\phi \left(Q_{i}\right)S}{\phi \left(Q_{i}\right)Z},\;i=1,\ldots ,N\;.$$

Here, $\phi \left(Q_{i}\right)$ interacts only with the fixed matrices S and Z, so each $y_{i}$ can be obtained independently in $O\left(d\right)$ time once S and Z are known. By reorganizing the computation in this way, linear attention eliminates the need to form and normalize an N×N attention matrix. Instead, it performs one linear pass to build S and Z, followed by a second linear pass to compute all $y_{i}$. This two-pass algorithm thus reduces both time and memory complexity from $O\left(N^{2}\right)$ to $O\left(N\right)$, while fully preserving the ability to model global context.

Inspired by Mamba, the MILA leverages linear attention and replaces the input-dependent forget gate with a fixed positional encoding mechanism. Instead of dynamically computing a forget gate ${\tilde{A}}_{i}$, MILA uses a fixed positional encoding $\Delta_{j}\in {\mathbb{R}}^{1\times d}$, such as LePE [38] or RoPE [39], to inject relative position information into each key:

(10) $$K_{j}\leftarrow K_{j}+\Delta_{j}.$$

The modified attention output is computed as:

(11) $$y_{i}=\frac{\phi \left(Q_{i}\right)\sum_{j=1}^{N}\phi {\left(K_{j}+\Delta_{j}\right)}^{T}V_{j}}{\phi \left(Q_{i}\right)\sum_{j=1}^{N}\phi {\left(K_{j}+\Delta_{j}\right)}^{T}},\;i=1,\ldots ,N.$$

This enables fully parallelized computation while preserving positional sensitivity. This substitution of a dynamic gate with a static positional encoding not only eliminates the need for recurrent or input-dependent gating but also ensures that positional information is uniformly encoded across all tokens in a single forward pass.

Inspired by Mamba’s structured block design, the MILT block adopts a modular architecture that efficiently fuses global semantic context with local spatial details. As illustrated in Figure 4, the MILT block first applies a depth-wise convolution and subsequent layer normalization to the input $X_{in}\in {\mathbb{R}}^{N\times C}$, producing the intermediate feature map $\hat{X}$. The computational workflow is then given by:

(12) $$\hat{X}=LN\left(X_{in}+DWConv\left(X_{in}\right)\right),$$

where $X_{in}$ is the input feature, and $DWConv\left(\cdot \right)$ and $LN\left(\cdot \right)$ refer to the depth-wise convolution and layer normalization operations, respectively. The normalized feature map $\hat{X}$ is projected via three learnable linear transformations:

(13) $$Q=\hat{X}W_{Q},\;K=\hat{X}W_{K},\;V=\hat{X}W_{V},\Delta =RoPE\left(\hat{X}\right),$$

where $W_{Q},W_{K},W_{V}\in {\mathbb{R}}^{C\times d}$ and $\Delta_{j}\in {\mathbb{R}}^{1\times d}$ is a fixed positional encoding. To linearize softmax, each query and augmented key is mapped via ϕ:${\tilde{Q}}_{i}=\phi \left(Q_{i}\right)$ and ${\tilde{K}}_{j}=\phi \left(K_{j}+\Delta_{j}\right)$. Two global accumulators are then computed as:

(14) $$\begin{array}{l} S=\sum_{j=1}^{N}{\tilde{K}}_{j}^{T}V_{j},\\ Z\;=\sum_{j=1}^{N}{\tilde{K}}_{j}^{T}, \end{array}$$

which aggregate all key-value and key contributions. The attention output for each position i is then computed in $O\left(d\right)$ as:

(15) $$A_{i}=\frac{{\tilde{Q}}_{i}S}{{\tilde{Q}}_{i}Z},\;i=1,\ldots ,N.$$

The resulting attention output is then processed by a gated subbranch, which employs a gated activation. The computation is as follows:

(16) $$\begin{array}{l} G=\sigma \left({\hat{X}W}_{\;G}\right),\\ \tilde{A}=A⊙G, \end{array}$$

where $W_{G}\in {\mathbb{R}}^{C\times C}$, $\sigma \left(\cdot \right)$ is SiLU activation, and ⊙ denotes element-wise multiplication. The gated output $\tilde{A}$ is subjected to a linear transformation to produce the MILA output $\hat{A}$, which is subsequently combined with the original input X via a residual connection to yield the enhanced feature Y. Finally, a layer normalization followed by a feed-forward network, combined with a residual connection, produces the final output of the MILT block, as illustrated below:

(17) $$X_{out}=FFN\left(LN\left(Y\right)\right)+Y,$$

where $FFN\left(\cdot \right)$ denotes the feed-forward neural network operation, and $X_{out}$ denotes the final output of the MILT block.

## 3. Experiments

### 3.1. Dataset

We evaluate the performance of the proposed approach for ultrasound breast tumor classification on the BUSI [40], BUS [41], and GDPH_SYSUCC [42] datasets.

The BUSI dataset comprises 780 ultrasound images acquired in 2018 at Baheya Women’s Cancer Early Detection and Treatment Hospital in Cairo, Egypt, from 600 female patients aged 25–75. All images are stored in PNG format at approximately 500 × 500 pixels and span three categories: malignant, benign and normal. In this study, only the benign and malignant categories from the BUSI dataset are utilized, comprising a total of 697 images, with 487 labeled as benign and 210 as malignant.

The BUS dataset contains 163 ultrasound images, of which 53 depict malignant tumors and 110 benign lesions. These scans were acquired at the UDIAT Diagnostic Center of Parc Taulí University Hospital in Sabadell, Spain on a Siemens ACUSON Sequoia C512 system and annotated by experienced radiologists.

The GDPH_SYSUCC dataset comprises 2405 breast ultrasound images drawn from two hospitals and annotated with BI-RADS scores via the Picture Archiving and Communication System. Scans were acquired on multiple platforms (Supersonic Aixplorer, Toshiba Aplio 500, Mindray DC-80, and Hitachi Ascendus) and labeled benign or malignant according to biopsy or surgical pathology. The collection includes 886 benign images from 526 patients and 1519 malignant images from 676 patients. Image resolutions range from 278 × 215 to 1280 × 800 pixels.

**Figure 5:**
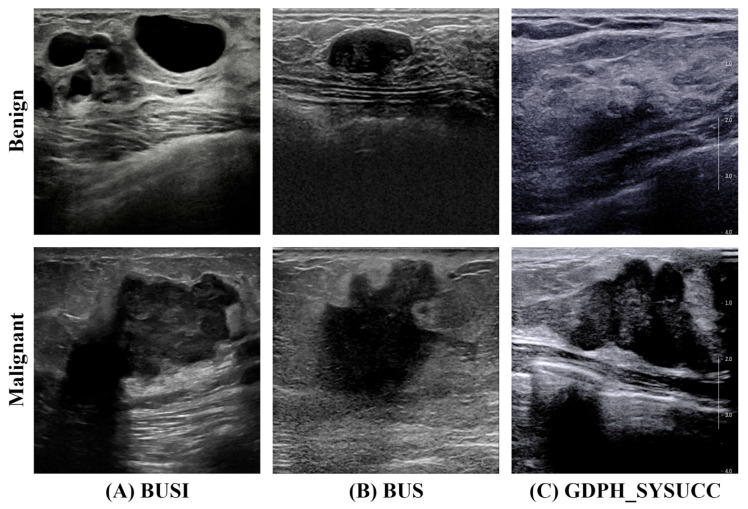
The samples of the three datasets.

Table 1 and Figure 5 provide a clearer overview of the class-wise image distribution and representative examples of the dataset, respectively, facilitating a better understanding of the data characteristics used in this study.

### 3.2. Training Details

The method is implemented in Python 3.10 using the PyTorch 2.7 framework on a workstation equipped with an NVIDIA RTX4070 GPU, an Intel Core i7-14700KF CPU, and 64GB of RAM.

The model employs a cross-domain segmentation prior to guide the classification backbone for breast tumor diagnosis. Accordingly, the first stage of deployment trains the segmentation branch on the non-classification BUSBRA dataset [43]. During this stage, automatic data augmentation is applied and all input images are resized to 256 × 256 pixels. Optimization is performed using the Adam optimizer with a weight-decay of 1 × 10^−4^. The branch is trained for 300 epochs with a batch size of 4, beginning at a learning rate of 1 × 10^−4^ that decays according to a cosine annealing schedule. Binary Cross-Entropy combined with Dice loss (BCEDice) serves as the objective function. The checkpoint exhibiting the highest validation accuracy is retained for downstream use. In the subsequent classification stage, these pretrained segmentation weights are loaded into the segmentation branch and remain frozen while training the classification backbone.

In the classification stage, the pretrained segmentation branch is first loaded and its weights are held constant to preserve the learned cross-domain priors. All inputs to the dual-stream model are uniformly resized to 256 × 256 pixels. Each original ultrasound image is fed through the frozen segmentation branch to extract multi-level feature maps, which are employed as segmentation priors by the classification backbone. The backbone network is initialized with random weights and optimized using Adam with a weight decay of 1 × 10^−4^. Training proceeds for 100 epochs with a batch size of 32. An initial learning rate of 1 × 10^−4^ is scheduled to decay according to cosine annealing, enabling fine-grained adjustments during later iterations. The classification loss is computed via categorical cross-entropy, and model checkpoints are saved at each epoch if the validation accuracy improves. Following completion of training, the checkpoint yielding the highest validation accuracy is selected for final testing.

### 3.3. Evaluation Metrics

The breast tumor classification performance is evaluated using five standard metrics: Accuracy (ACC), Recall, Weighted Precision (Pre_w_), Weighted F1-score (F1_w_), and Cohen’s Kappa coefficient (Kappa). These measures together deliver a comprehensive assessment of classification effectiveness, resilience to class imbalance, and agreement beyond chance. The formal definitions of each metric are provided below:

(18) $$\begin{array}{l} ACC=\frac{TP+TN}{TP+TN+FP+FN},\\ Recall=\frac{TP}{TP+FN},\\ {Pre}_{W}=\sum_{i=1}^{C}w_{i}\cdot \frac{TP_{i}}{TP_{i}+FP_{i}},\\ F1_{W}=\sum_{i=1}^{C}w_{i}\cdot \frac{2\cdot {Precision}_{i}\cdot {Recall}_{i}}{{Precision}_{i}+{Recall}_{i}},\\ Kappa=\frac{ACC-p_{e}}{1-p_{e}},p_{e}=\sum_{i=1}^{C}\frac{n_{i+}\cdot n_{+i}}{N^{2}}, \end{array}$$

where TN, TP, FN, and FP denote true negatives, true positives, false negatives, and false positives, respectively. C denotes the total number of categories, $w_{i}$ is the fraction of samples belonging to category i, and $TP_{i}$ and $FP_{i}$ are the counts of true positives and false positives for category i, respectively. $n_{i+}$ and $n_{+1}$ denote the sum of the ith row and the ith column of the confusion matrix, and N represents the total sample size.

A stratified five-fold cross-validation scheme is employed throughout all experiments to provide an unbiased assessment of our method. Specifically, the dataset is partitioned into five equal folds; in each iteration, four folds serve as the training set and the remaining fold as the test set. This procedure is repeated five times, ensuring that every sample is used for validation exactly once, thereby reducing overfitting and delivering consistent performance estimates across varying data splits. We compare our model with several representative and competitive architectures to demonstrate the superiority of our DSDNet, including ConvNeXt [44] proposed in 2022, Focal [45] introduced in 2022, UniFormer [46] and BiFormer [47] released in 2023, TransNeXt [48] published in 2024, and the recently proposed MambaOut [49] in 2025.

## 4. Results and Discussion

### 4.1. Comparison with the State-of-the-Art-Methods

#### 4.1.1. Quantitative Comparison

All models are trained and evaluated under an identical five-fold cross-validation protocol on three benchmark breast ultrasound datasets: BUSI, BUS and GDPH_SYSUCC. We measure ACC, Recall, Pre_w_, F1_w_ and Kappa. Detailed numerical results appear in Table 2, Table 3 and Table 4, with the best score in each column highlighted in bold.

Table 2 reports the classification results on the BUSI dataset, comparing our DSDNet against six state-of-the-art backbones. DSDNet achieves an ACC of 0.878, Recall of 0.866, Pre_w_ of 0.880, F1_w_ of 0.878 and Kappa of 0.724, all of which are the highest among the evaluated methods. In particular, its Kappa exceeds that of the next best model, BiFormer-S, by over 0.05, indicating substantially better agreement with ground truth labels. These results demonstrate that the integration of cross-domain segmentation priors and specialized attention modules in DSDNet affords more precise delineation of tumor regions and more robust suppression of speckle noise than existing architectures under moderate training sample sizes.

Table 3 highlights the classification performance of DSDNet on the BUS dataset, where it achieves the highest scores across all metrics: ACC of 0.836, Recall of 0.789, Pre_w_ of 0.839, F1_w_ of 0.828, and Kappa of 0.606, outperforming six state-of-the-art methods, including BiFormer-S with a Kappa of 0.569 and TransNeXt-T with a Kappa of 0.518. The superior performance of DSDNet underscores its robustness in handling the limited sample size of the BUS dataset, leveraging its cross-domain segmentation priors to guide the classification backbone toward capturing global tumor morphology, while the DSMA and SPGF modules enhance lesion mask representations and feature fusion, ensuring stable and accurate diagnosis even under challenging imaging conditions.

Table 4 demonstrates that on the multi-center GDPH_SYSUCC dataset, DSDNet not only achieves ACC of 0.882, Recall of 0.878, Pre_w_ of 0.884 and Kappa of 0.749, but also delivers the highest F1_w_ of 0.883, surpassing both MambaOut-B and BiFormer-S and reflecting its superior balance between precision and sensitivity across diverse imaging conditions. The superior performance of DSDNet highlights its robustness in handling the diverse, multi-center GDPH_SYSUCC dataset. The MILT block, a core feature extraction module in DSDNet, enhances this capability by integrating the MILA to delineate low-contrast tumor boundaries and capture complex anatomical structures, contributing to a significant Kappa improvement of 0.04 over MambaOut-B. Additionally, the DSMA and SPGF modules amplify salient tumor features and ensure effective prior integration, solidifying DSDNet’s stability and accuracy in ultrasound breast tumor diagnosis.

Across datasets of varying scale and domain diversity, DSDNet consistently secures top ACC, Recall, Pre_w_, F1_w_ and Kappa. Its decoupled dual-stream architecture ensures stable spatial guidance, and its three specialized modules sharpen boundary detection, suppress background artifacts and integrate global and local context. The result is a robust, high-accuracy ultrasound breast tumor diagnosis model suitable for all testing scenarios.

#### 4.1.2. Qualitative Visualization

**Figure 6:**
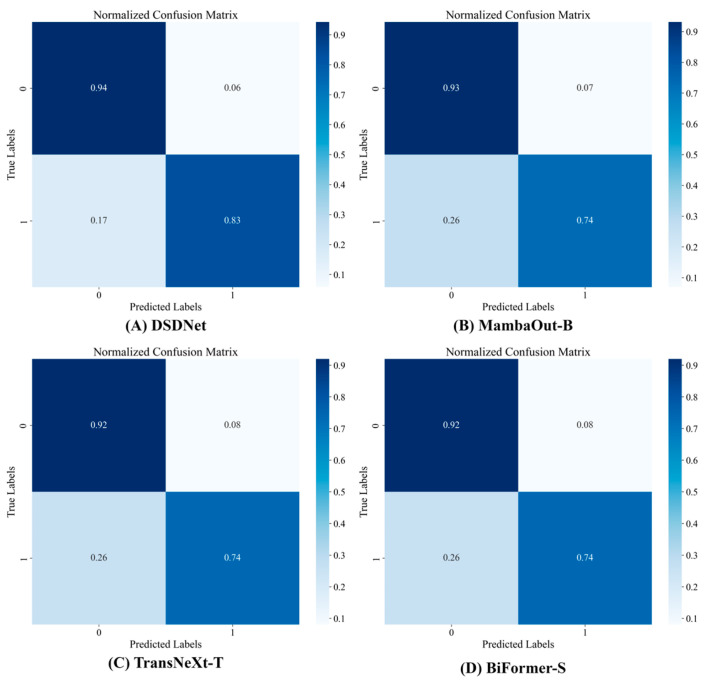
Confusion matrix of breast tumor classification on the BUSI dataset.

**Figure 7:**
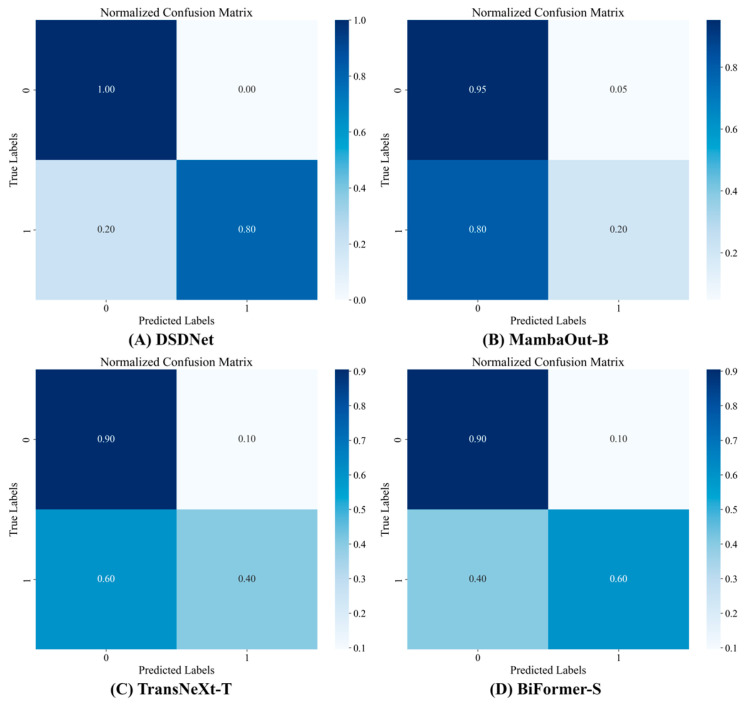
Confusion matrix of breast tumor classification on the BUS dataset.

**Figure 8:**
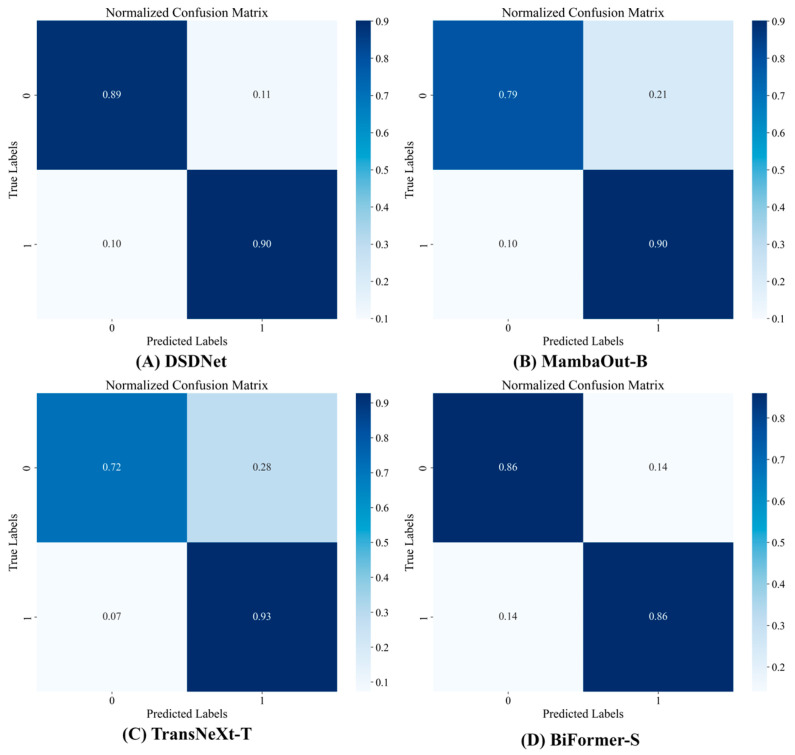
Confusion matrix of breast tumor classification on the GDPH_SYSUCC dataset.

**Figure 9:**
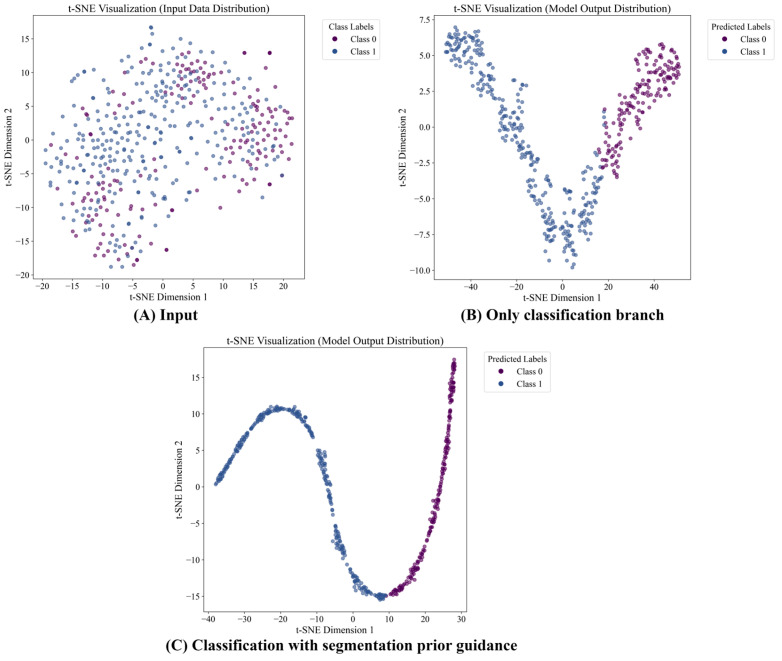
Visualization of ablation for segmentation prior guidance on the GDPH_SYSUCC dataset in t-SNE space.

To qualitatively evaluate classification behavior under the inherent challenges of breast ultrasound imaging, we employ normalized confusion matrices to visualize per-class prediction distributions. Figure 6, Figure 7, Figure 8 and Figure 9 compare four high-performing models, including DSDNet, MambaOut-B, TransNeXt-T and BiFormer-S, on the BUSI, BUS and GDPH_SYSUCC datasets. By inspecting these matrices, we can intuitively assess the ability of each architecture to distinguish benign from malignant cases, pinpoint common error patterns, and highlight how DSDNet’s cross-domain segmentation priors combined with the DSMA, SPGF and MILT modules lead to more balanced, accurate predictions across all classes.

From Figure 6 it can be seen that on the BUSI dataset DSDNet achieves 94% benign recall with only 6% benign false negatives, substantially outperforming MambaOut-B, which misclassifies 26% of malignant cases, while TransNeXt-T and BiFormer-S exhibit pronounced class imbalance as reflected by the 26% benign false-positive rate in TransNeXt-T. From Figure 7 it becomes clear that in the BUS dataset DSDNet attains perfect benign classification at 100% while limiting malignant false negatives to 20%, whereas TransNeXt-T and BiFormer-S incur malignant miss rates of 60% and 40%, respectively. From Figure 8 it is evident that on the GDPH_SYSUCC dataset DSDNet achieves 89% recall for benign lesions and 90% recall for malignant lesions, limiting misclassification in either direction to roughly 10%. MambaOut-B matches DSDNet’s malignant recall of 90% but sacrifices benign recall of 79%, leading to a 21% over-diagnosis rate for benign cases. TransNeXt-T attains the highest malignant recall of 93% yet incurs the largest benign false-negative rate of 28%, while BiFormer-S delivers symmetric but lower recalls of 86% for both classes. Collectively, these results underline DSDNet’s superior balance between sensitivity and specificity on the GDPH_SYSUCC dataset.

From the normalized confusion matrices across all datasets, a few common trends emerge. DSDNet consistently outperforms other models, with high true positive rates and a low number of false positives, especially in detecting benign cases across all three datasets. Its architecture, which integrates cross-domain segmentation priors and attention modules, aids in distinguishing subtle differences between malignant and benign lesions. MambaOut-B performs well, especially in the benign class, but tends to have higher false positives in the malignant class compared to DSDNet. On the other hand, TransNeXt-T and BiFormer-S struggle more with false negatives and false positives in malignant case detection, particularly in datasets with more challenging images, like the GDPH_SYSUCC dataset. This analysis shows that while all models perform decently, DSDNet stands out due to its balanced predictions, reducing misclassifications across all classes and datasets. In the Supplementary Materials, Figures S1–S3 present the classification performance of DSDNet in comparison with ConvNeXt-B, Focal-B and UniFormer-B across all datasets via confusion matrices, which fully corroborates the aforementioned conclusions.

### 4.2. Ablation Studies

#### 4.2.1. Ablation of Segmentation Prior Guidance

To evaluate the impact of segmentation prior guidance on the ability of DSDNet to distinguish benign from malignant breast lesions, an ablation study is performed comparing the classification-only variant against the full dual-branch model incorporating segmentation priors.

Quantitative results in Table 5 show that adding segmentation prior raises ACC from 0.824 to 0.882, Recall from 0.797 to 0.878, Pre_w_ from 0.824 to 0.884, F1_w_ from 0.820 to 0.883, and Kappa from 0.610 to 0.749. These results demonstrate that segmentation priors offer significant spatial guidance, which enhances the ability of the model to identify and delineate tumor-related features in breast ultrasound images.

The t-SNE visualizations in Figure 9 further reinforce this conclusion. Without segmentation prior guidance (Figure 9B), the classification backbone produces relatively dispersed output features, leading to weaker inter-class separability. In contrast, with segmentation prior integration (Figure 9C), the output embeddings exhibit a clearer margin and more compact intra-class distributions, indicating more discriminative representations.

Together, these quantitative and qualitative results validate the effectiveness of segmentation prior guidance in improving DSDNet’s robustness and accuracy for ultrasound breast tumor diagnosis. Table S1 in the Supplementary Materials, which reports the ablation results for segmentation prior guidance on the hybrid dataset, also corroborates this finding.

#### 4.2.2. Ablation of SPGF and DSMA Modules

**Figure 10:**
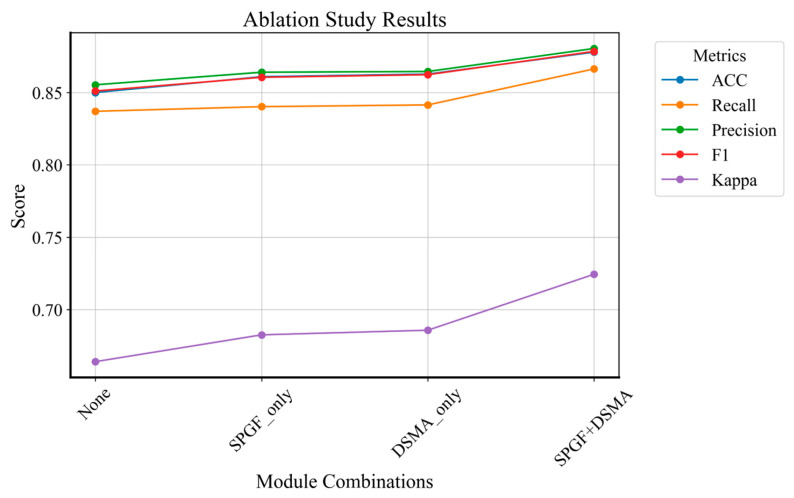
Visualization of ablation for SPGF and DSMA modules on the BUSI dataset.

To validate that the DSMA and SPGF modules enable DSDNet to effectively leverage cross-domain segmentation priors for guiding ultrasound breast tumor classification, we conduct an ablation study in which these modules are replaced by standard convolutional blocks to establish the baseline. Table 6 provides the performance of various module configurations, while Figure 10 illustrates the ablation outcomes for a more intuitive comparison. Table S2 in the Supplementary Materials presents the ablation results for the SPGF and DSMA modules on the hybrid dataset.

Table 6 quantifies how each component contributes to diagnostic performance on the BUSI dataset. Replacing both SPGF and DSMA with plain convolutions yields the lowest ACC of 0.850, Recall of 0.837, Pre_w_ of 0.855, F1_w_ of 0.851, and Kappa of 0.664. Introducing the SPGF module alone raises ACC to 0.861, Recall to 0.840, Pre_w_ to 0.864, F1_w_ to 0.861, and Kappa to 0.683. The DSMA module alone increases ACC to 0.863, Recall to 0.841, Pre_w_ to 0.865, F1_w_ to 0.862, and Kappa to 0.686. When both the SPGF and DSMA modules are integrated, DSDNet achieves the highest performance across all metrics. This highlights the synergistic effect of the SPGF and DSMA modules. Specifically, SPGF enhances the network by fusing spatial priors from the segmentation stream, which provides rich contextual information for accurately capturing the global tumor morphology. Meanwhile, DSMA refines the segmentation priors by selectively focusing on foreground tumor regions while suppressing irrelevant background noise. The combination of these two modules enables DSDNet to better leverage the robust cross-domain priors from the frozen segmentation branch, ultimately enhancing the diagnostic accuracy and stability across diverse datasets.

Figure 10 visualises these improvements across all metrics as the model evolves from the baseline to SPGF, DSMA, and finally SPGF + DSMA. Each metric traces an upward trajectory, with the most pronounced gain observed in Kappa, indicating enhanced agreement beyond chance. Both the table and the plot converge on a single conclusion: the combined SPGF and DSMA modules are essential for fully exploiting cross-domain segmentation priors in breast ultrasound classification.

#### 4.2.3. Ablation of MILA Within the MILT Block

**Figure 11:**
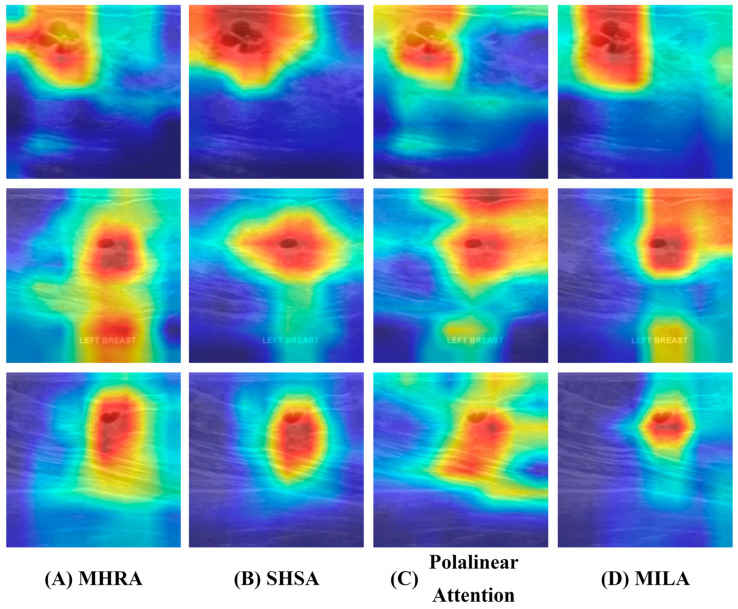
Visualization of feature maps from the MILT block on the BUSI dataset.

To evaluate the contribution of the MILA module to improved delineation of tumor boundaries and extraction of complex anatomical features in the DSDNet model, an ablation study is conducted within the MILT block. Three alternative attention schemes: MHRA [46], SHSA [50] and Polalinear Attention [51], are substituted for the MILA module and assessed under identical conditions. Quantitative results appear in Table 7 and Figure 11 presents heatmap visualizations of feature maps produced by the classification backbone. These comparisons demonstrate that the MILA achieves more accurate highlighting of tumor margins and heterogeneous tissue structures in breast ultrasound images.

Table 7 compares the effect of replacing the MILA module with three alternative attention schemes: MHRA, SHSA and Polalinear Attention, under identical experimental conditions. On the BUSI dataset, the MILA module delivers superior results for every metric. ACC increases from values ranging between 0.869 and 0.872 up to 0.878. Recall climbs from a range of 0.835 to 0.855 to 0.866. Pre_w_ rises from 0.865 to 0.875 to 0.880. F1_w_ moves from 0.861 to 0.871 to 0.878. Kappa improves from 0.682 to 0.706 to 0.724. These consistent gains demonstrate that the MILA module achieves greater sensitivity to true tumor regions and stronger agreement with ground truth, confirming its enhanced ability to outline tumor margins and capture heterogeneous anatomical patterns in breast ultrasound images. The performance comparison of MILA with other attention mechanisms on the hybrid dataset is presented in Table S3 of the Supplementary Materials.

The heatmaps in Figure 11 show that MHRA, SHSA and Polalinear Attention produce broad, uneven activations extending into surrounding tissue, whereas the proposed linear attention module generates sharply focused response regions that coincide with actual tumor boundaries and minimize background noise. This behavior reflects effective positional encoding and local feature refinement within the module and aligns with its superior quantitative performance in lesion delineation and localization.

### 4.3. Cross-Dataset Validation

**Figure 12:**
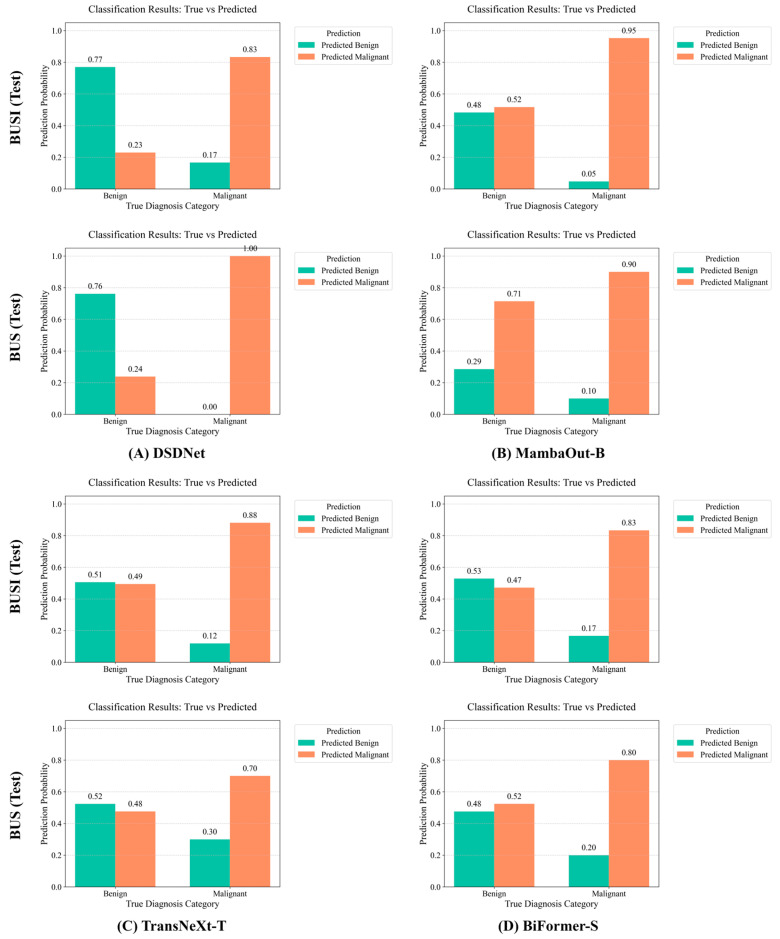
Visualization of classification performance under cross-dataset validation.

To assess the generalization ability conferred by pretraining the segmentation branch on cross-domain data, we perform a cross-dataset validation. In this experiment, models trained exclusively on the GDPH_SYSUCC dataset are directly evaluated, without any fine-tuning, on the BUSI and BUS datasets, enabling us to quantify the robustness of our segmentation-guided framework under heterogeneous imaging conditions. Table 8 presents the quantitative classification results under cross-dataset validation, while Figure 12 provides a corresponding visualization of the performance differences across the BUSI and BUS datasets.

The results presented in Table 8 demonstrate that DSDNet outperforms other methods across both test sets. Specifically, when trained on the GDPH_SYSUCC dataset and tested on the BUSI dataset, DSDNet achieves an ACC of 0.737, Recall of 0.724, Pre_w_ of 0.753, F1_w_ of 0.742 and Kappa of 0.428. These results outperform UniFormer-B, which reaches 0.621 ACC and 0.685 Recall, BiFormer-S with 0.619 ACC and 0.664 Recall, and all other models by substantial margins. When tested on the BUS dataset, DSDNet again leads with 0.768 ACC, 0.758 Recall, 0.787 Pre_w_, 0.770 F1_w_ and 0.494 Kappa, maintaining an advantage of approximately 11 points in F1_w_ and 20 points in Kappa over UniFormer-B and larger gaps against TransNeXt-T and MambaOut-B. These results highlight the robustness of the segmentation-guided framework, as DSDNet effectively generalizes across different clinical centers and datasets. The improvements in performance, particularly in comparison to other models such as ConvNeXt-B and BiFormer-S, demonstrate the effectiveness of the proposed approach in leveraging cross-domain segmentation priors for consistent and accurate breast tumor classification.

In Figure 12, true versus predicted class probabilities for DSDNet and three strong models, including MambaOut-B, TransNeXt-T, and BiFormer-S, are depicted as bar charts for both the BUSI and BUS datasets. As shown in Figure 12, DSDNet achieves high and balanced accuracy for both benign and malignant cases in cross-dataset validation on BUSI and BUS, avoiding the bias toward malignant detection seen in other models that often misclassify benign lesions as malignant and thereby compromise diagnostic performance. These findings demonstrate that cross-domain segmentation prior guidance significantly enhances generalization. DSDNet is capable of handling multi-center data without overfitting to any single domain.

### 4.4. Complexity Analysis

In this section, we conduct a detailed analysis of computational requirements of DSDNet by measuring its total parameter count, GFLOPs, and inference time. Evaluating these metrics is essential to ensure that the model can be deployed effectively in clinical settings with limited computational resources.

**Figure 13:**
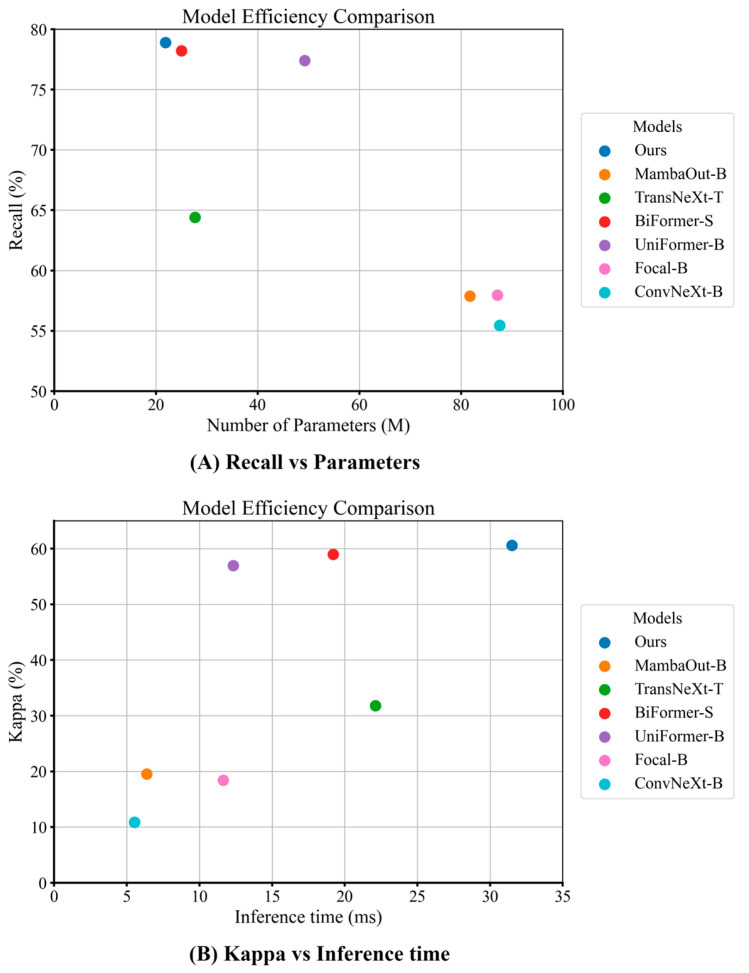
Model efficiency comparison on the BUS dataset: Recall vs. Parameters and Kappa vs. Inference time.

Table 9 shows that DSDNet achieves the smallest parameter footprint among all evaluated models thanks to its streamlined classification backbone. However, its dual-stream design, which incorporates a frozen segmentation branch to supply cross-domain spatial priors, results in higher GFLOPs and longer inference time. Figure 13 further illustrates these trade-offs on the BUS dataset: despite having the fewest parameters, DSDNet attains the highest Recall, and although its inference time is relatively large, it secures the best Kappa.

This analysis highlights deliberate performance efficiency compromise between DSDNet and underscores its key innovation: leveraging frozen segmentation priors for robust diagnosis. To achieve a more favorable trade-off in future work, we plan to adopt a more efficient segmentation model for the prior branch and streamline the classification backbone so as to preserve dual-stream benefits while reducing computational overhead.

## 5. Conclusions

This paper presents a novel cross-domain segmentation prior guided classification strategy for robust ultrasound breast tumor diagnosis, realized through the DSDNet. DSDNet adopts a decoupled dual-stream architecture, where a frozen segmentation branch provides cross-domain spatial priors to guide the classification backbone. To enhance the effectiveness of this framework, three key modules are proposed: (1) the DSMA module, which strengthens lesion representations by modeling foreground-background dependencies; (2) the SPGF module, which adaptively integrates multi-scale priors into backbone features to improve morphological understanding; and (3) the MILT block, built upon the MILA mechanism, which enables efficient extraction of global context and fine-grained tumor boundaries via a streamlined linear attention design. Together, these innovations enable DSDNet to achieve accurate and generalizable classification across heterogeneous imaging conditions.

Comprehensive experiments on the BUSI, BUS, and GDPH_SYSUCC datasets validate the effectiveness of the proposed DSDNet for ultrasound breast tumor diagnosis. By explicitly integrating cross-domain segmentation priors into the classification process, DSDNet effectively alleviates common challenges in breast ultrasound imaging, such as speckle noise, low lesion-to-background contrast, and large variations in tumor morphology. The dual-stream design enables the model to maintain stable performance across data acquired from different institutions and imaging devices, demonstrating a clear advantage in robustness and generalization under domain shifts.

Despite these advantages, DSDNet also has several limitations. The introduction of a frozen segmentation branch increases computational complexity, leading to higher GFLOPs and longer inference time compared with single-stream classification models. In addition, the current framework focuses on binary classification of benign and malignant tumors and does not explicitly model normal breast tissue, which limits its applicability in comprehensive clinical screening scenarios.

Future work will therefore focus on several directions. First, we plan to replace the current segmentation branch with a more lightweight yet effective architecture to reduce computational overhead while preserving the benefits of segmentation priors. Second, further simplification and optimization of the classification backbone will be explored to improve efficiency. Finally, we aim to extend the dataset to include normal breast ultrasound images, enabling the development of a more complete diagnostic system capable of distinguishing healthy, benign, and malignant cases, thereby enhancing the clinical relevance of the proposed approach.

## Figures and Tables

**Table 1:** Class Distribution of BUSI, BUS, and GDPH_SYSUCC Datasets.

Category	BUSI	BUS	GDPH_SYSUCC
	Number of Images	Number of Images	Number of Images
Benign (Class 0)	487	110	886
Malignant (Class 1)	210	53	1519
Total	697	163	2405

**Table 2:** Classification performance comparison on the BUSI dataset.

Methods	ACC	Recall	Pre_w_	F1_w_	Kappa
ConvNeXt-B	0.811 ± 0.030	0.771 ± 0.028	0.808 ± 0.030	0.809 ± 0.029	0.558 ± 0.064
Focal-B	0.782 ± 0.050	0.726 ± 0.062	0.776 ± 0.055	0.775 ± 0.053	0.475 ± 0.126
UniFormer-B	0.841 ± 0.022	0.799 ± 0.032	0.843 ± 0.020	0.836 ± 0.023	0.622 ± 0.054
BiFormer-S	0.858 ± 0.009	0.829 ± 0.019	0.857 ± 0.010	0.856 ± 0.010	0.670 ± 0.025
TransNeXt-T	0.858 ± 0.015	0.842 ± 0.019	0.860 ± 0.015	0.858 ± 0.015	0.678 ± 0.034
MambaOut-B	0.847 ± 0.014	0.815 ± 0.016	0.847 ± 0.012	0.845 ± 0.013	0.644 ± 0.028
DSDNet (Ours)	**0.878 ±** **0.028**	**0.866 ±** **0.034**	**0.880 ±** **0.029**	**0.878 ±** **0.027**	**0.724 ±** **0.063**

**Table 3:** Classification performance comparison on the BUS dataset.

Methods	ACC	Recall	Pre_w_	F1_w_	Kappa
ConvNeXt-B	0.687 ± 0.026	0.555 ± 0.099	0.578 ± 0.177	0.589 ± 0.085	0.109 ± 0.184
Focal-B	0.706 ± 0.039	0.580 ± 0.072	0.662 ± 0.140	0.637 ± 0.082	0.184 ± 0.159
UniFormer-B	0.816 ± 0.047	0.774 ± 0.052	0.813 ± 0.049	0.812 ± 0.048	0.569 ± 0.108
BiFormer-S	0.828 ± 0.019	0.782 ± 0.047	0.835 ± 0.025	0.821 ± 0.026	0.590 ± 0.066
TransNeXt-T	0.742 ± 0.045	0.644 ± 0.089	0.710 ± 0.153	0.697 ± 0.095	0.318 ± 0.188
MambaOut-B	0.718 ± 0.033	0.579 ± 0.050	0.716 ± 0.159	0.641 ± 0.064	0.195 ± 0.122
DSDNet (Ours)	**0.836 ±** **0.078**	**0.789 ±** **0.098**	**0.839 ±** **0.077**	**0.827 ±** **0.085**	**0.606 ±** **0.195**

**Table 4:** Classification performance comparison on the GDPH_SYSUCC dataset.

Methods	ACC	Recall	Pre_w_	F1_w_	Kappa
ConvNeXt-B	0.830 ± 0.016	0.826 ± 0.013	0.835 ± 0.013	0.832 ± 0.015	0.641 ± 0.030
Focal-B	0.717 ± 0.025	0.672 ± 0.033	0.716 ± 0.020	0.705 ± 0.029	0.361 ± 0.061
UniFormer-B	0.870 ± 0.015	0.865 ± 0.016	0.872 ± 0.014	0.871 ± 0.015	0.724 ± 0.031
BiFormer-S	0.871 ± 0.013	0.867 ± 0.012	0.873 ± 0.012	0.871 ± 0.013	0.725 ± 0.026
TransNeXt-T	0.866 ± 0.023	0.857 ± 0.029	0.868 ± 0.023	0.866 ± 0.024	0.712 ± 0.051
MambaOut-B	0.864 ± 0.011	0.856 ± 0.011	0.865 ± 0.010	0.864 ± 0.011	0.709 ± 0.022
DSDNet (Ours)	**0.882 ±** **0.014**	**0.878 ±** **0.017**	**0.884 ±** **0.015**	**0.883 ±** **0.014**	**0.749 ±** **0.031**

**Table 5:** Ablation results for segmentation prior guidance on the GDPH_SYSUCC dataset.

Segmentation Branch	Classification Branch	ACC	Recall	Pre_w_	F1_w_	Kappa
×	√	0.824 ± 0.010	0.797 ± 0.020	0.824 ± 0.009	0.820 ± 0.012	0.610 ± 0.029
√	√	**0.882 ±** **0.014**	**0.878 ±** **0.017**	**0.884 ±** **0.015**	**0.883 ±** **0.014**	**0.749 ±** **0.031**

**Table 6:** Ablation results for SPGF and DSMA modules on the BUSI dataset.

SPGF	DSMA	ACC	Recall	Pre_w_	F1_w_	Kappa
×	×	0.850 ± 0.018	0.837 ± 0.008	0.855 ± 0.009	0.851 ± 0.016	0.664 ± 0.029
√	×	0.861 ± 0.024	0.840 ± 0.023	0.864 ± 0.020	0.860 ± 0.023	0.683 ± 0.049
×	√	0.863 ± 0.023	0.841 ± 0.023	0.864 ± 0.023	0.862 ± 0.022	0.686 ± 0.050
√	√	**0.878 ±** **0.028**	**0.866 ±** **0.034**	**0.880 ±** **0.029**	**0.878 ±** **0.027**	**0.724 ±** **0.063**

**Table 7:** Performance comparison of MILA and other attention mechanisms on the BUSI dataset.

Methods	ACC	Recall	Pre_w_	F1_w_	Kappa
MHRA	0.869 ± 0.015	0.854 ± 0.022	0.871 ± 0.016	0.869 ± 0.015	0.703 ± 0.035
SHSA	0.872 ± 0.018	0.853 ± 0.036	0.875 ± 0.021	0.871 ± 0.019	0.706 ± 0.048
Polalinear Attention	0.862 ± 0.022	0.835 ± 0.025	0.865 ± 0.020	0.861 ± 0.021	0.681 ± 0.046
MILA	**0.878 ±** **0.028**	**0.866 ±** **0.034**	**0.880 ±** **0.029**	**0.878 ±** **0.027**	**0.724 ±** **0.063**

**Table 8:** Classification performance comparison under cross-dataset validation.

Methods	GDPH_SYSUCC(Training)/BUSI(Test)					GDPH_SYSUCC(Training)/BUS(Test)				
	ACC	Recall	Pre_w_	F1_w_	Kappa	ACC	Recall	Pre_w_	F1_w_	Kappa
ConvNeXt-B	0.655 ± 0.041	0.685 ± 0.037	0.727 ± 0.030	0.666 ± 0.041	0.321 ± 0.069	0.656 ± 0.062	0.590 ± 0.086	0.640 ± 0.076	0.642 ± 0.072	0.182 ± 0.171
Focal-B	0.525 ± 0.027	0.603 ± 0.022	0.679 ± 0.020	0.524 ± 0.030	0.160 ± 0.036	0.454 ± 0.096	0.545 ± 0.099	0.621 ± 0.156	0.429 ± 0.111	0.070 ± 0.149
UniFormer-B	0.621 ± 0.018	0.685 ± 0.019	0.748 ± 0.022	0.628 ± 0.019	0.300 ± 0.032	0.652 ± 0.119	0.670 ± 0.137	0.708 ± 0.122	0.662 ± 0.116	0.301 ± 0.241
BiFormer-S	0.617 ± 0.049	0.664 ± 0.048	0.717 ± 0.041	0.626 ± 0.049	0.274 ± 0.083	0.583 ± 0.084	0.614 ± 0.088	0.663 ± 0.081	0.593 ± 0.082	0.196 ± 0.154
TransNeXt-T	0.598 ± 0.044	0.658 ± 0.046	0.719 ± 0.044	0.605 ± 0.045	0.257 ± 0.077	0.613 ± 0.084	0.622 ± 0.061	0.670 ± 0.056	0.619 ± 0.079	0.224 ± 0.114
MambaOut-B	0.583 ± 0.039	0.656 ± 0.043	0.730 ± 0.046	0.585 ± 0.039	0.248 ± 0.069	0.503 ± 0.051	0.555 ± 0.038	0.620 ± 0.056	0.502 ± 0.064	0.089 ± 0.059
DSDNet (Ours)	**0.737 ±** **0.052**	**0.724 ±** **0.059**	**0.753 ±** **0.050**	**0.742 ±** **0.051**	**0.428 ±** **0.111**	**0.768 ±** **0.056**	**0.758 ±** **0.076**	**0.787 ±** **0.068**	**0.770 ±** **0.055**	**0.494 ±** **0.125**

**Table 9:** Comparison of model parameters, GFLOPs, and inference time.

Methods	Params (M)	GFLOPs	Inference Time (ms)
ConvNeXt-B	87.57	20.14	5.53
Focal-B	87.13	20.0	11.64
UniFormer-B	49.26	10.20	12.33
BiFormer-S	25.02	4.22	19.21
TransNeXt-T	27.68	7.35	22.11
MambaOut-B	81.75	20.72	6.37
DSDNet	21.88	98.64	31.50

## Data Availability

Data generated during the current study are available from the corresponding author on reasonable request. The source code of the proposed DSDNet is publicly available at https://github.com/jiyue828/DSDNet.git (accessed on 30 December 2025).

## Supplementary Materials

The following supporting information can be downloaded at: https://www.mdpi.com/article/10.3390/s26030974/s1, Figure S1: Confusion matrix of breast tumor classification on the BUSI dataset; Figure S2: Confusion matrix of breast tumor classification on the BUS dataset; Figure S3: Confusion matrix of breast tumor classification on the GDPH_SYSUCC dataset; Table S1: Ablation results for segmentation prior guidance on the hybrid dataset; Table S2: Ablation results for SPGF and DSMA modules on the hybrid dataset; Table S3: Performance comparison of MILA and other attention mechanisms on the hybrid dataset.
