# Supplementary material for: Robust Dual-Stream Diagnosis Network for Ultrasound Breast Tumor Classification with Cross-Domain Segmentation Priors
Source: Sensors (Basel). 2026 Feb 2;26(3):974. doi: 10.3390/s26030974 (PMC12899992; doi:10.3390/s26030974)
Supplement: Supplementary file 1 [file sensors-26-00974-s001.zip › sensors-4101001-supplementary.pdf]

A. Qualitative Visualization of the remaining models

As shown in Figures S1–S3, compared with the qualitative visualization results of the remaining models, DSDNet consistently demonstrates clearer and more reliable classification behavior across all datasets. The normalized confusion matrices show that DSDNet achieves a better balance between true positives and false positives, particularly in benign case identification, while maintaining strong performance on malignant cases. Benefiting from the integration of cross-domain segmentation priors and dedicated attention mechanisms, DSDNet more effectively distinguishes subtle lesion characteristics, resulting in fewer misclassifications than other competing models under challenging imaging conditions.

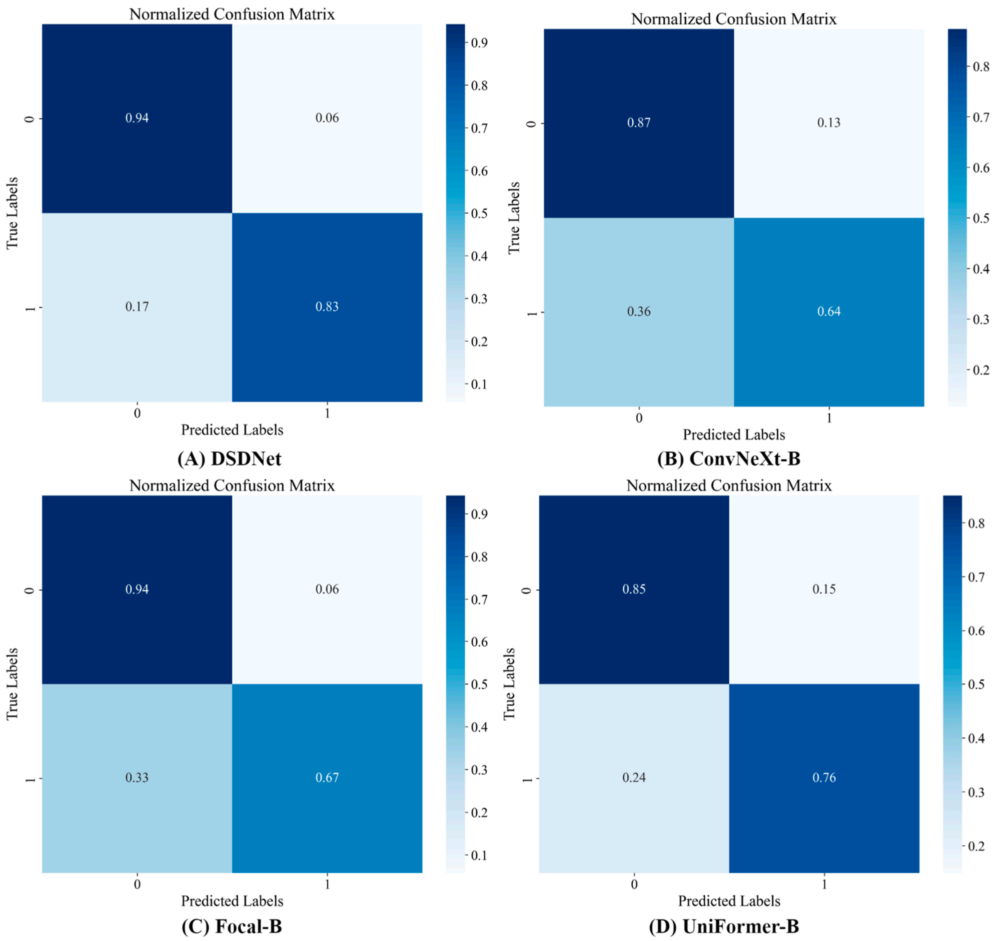

Figure S1. Confusion matrix of breast tumor classification on the BUSI dataset.

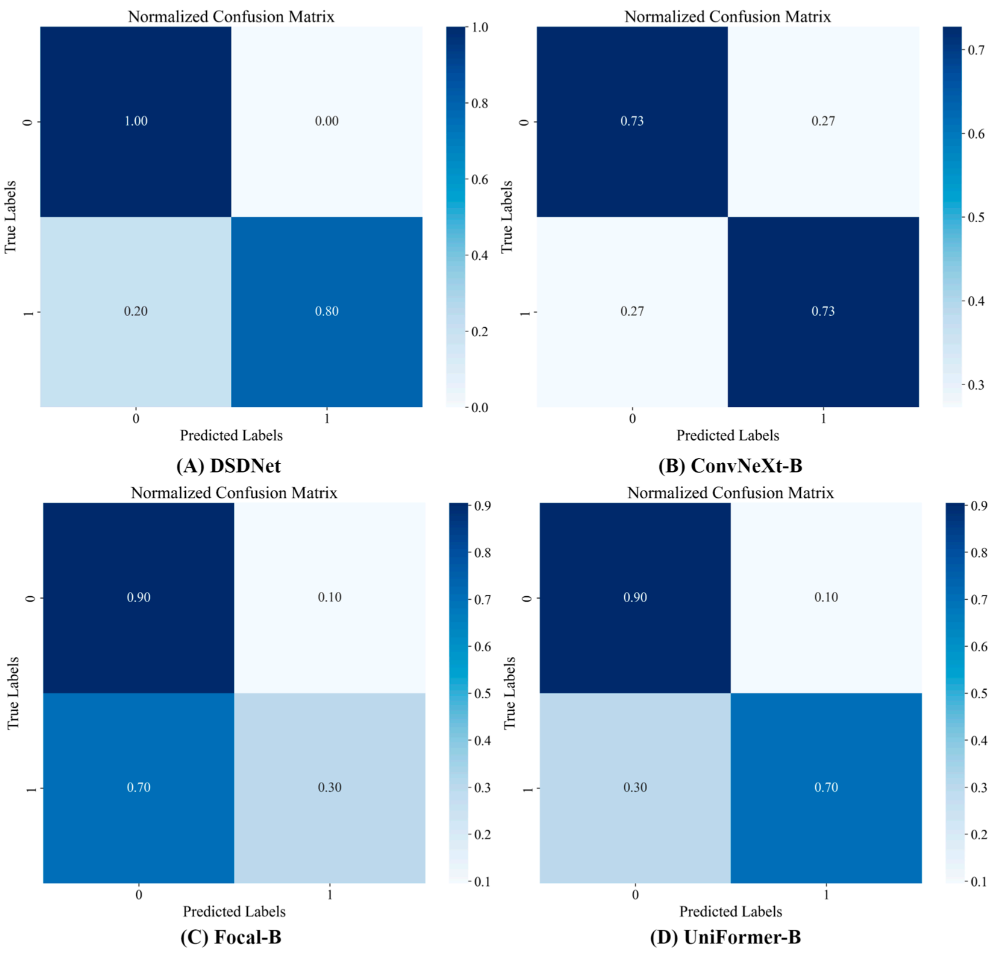

Figure S2. Confusion matrix of breast tumor classification on the BUS dataset.

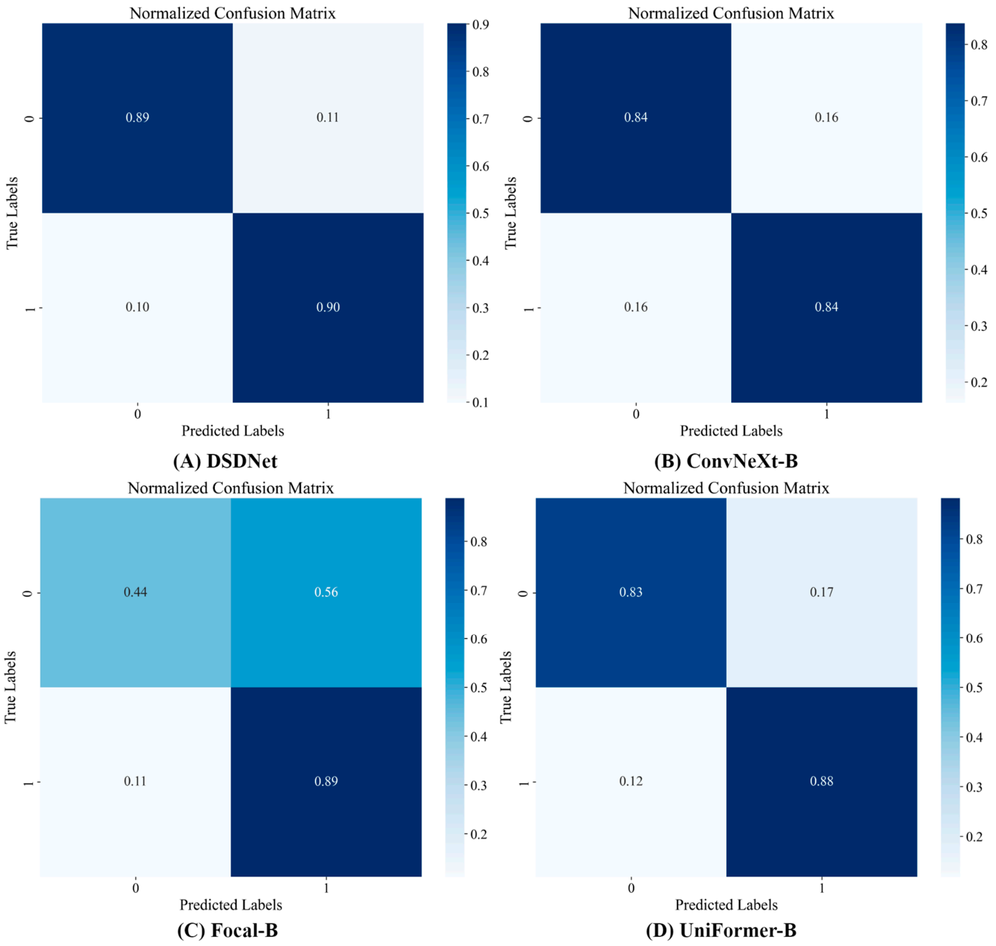

Figure S3. Confusion matrix of breast tumor classification on the GDPH_SYSUCC dataset.

B. Ablation of Segmentation Prior Guidance on the Hybrid Dataset

Table S1. Ablation results for segmentation prior guidance on the hybrid dataset.

Segmentation branch	Classification branch	ACC	Recall	Prew	F1w	Kappa
×	√	0.830	0.800	0.829	0.826	0.621
√	√	**0.907**	**0.888**	**0.906**	**0.906**	**0.786**

C. Ablation of SPGF and DSMA Modules on the Hybrid Dataset

Table S2. Ablation results for SPGF and DSMA modules on the hybrid dataset.

SPGF	DSMA	ACC	Recall	Pre_w	$F1_w$	Kappa
×	×	0.860	0.829	0.859	0.859	0.674
√	×	0.891	0.870	0.891	0.891	0.750
×	√	0.876	0.846	0.874	0.874	0.710
√	√	**0.907**	**0.888**	**0.906**	**0.906**	**0.786**

D. Ablation of MILA within the MILT Block on the Hybrid Dataset

Table S3. Performance comparison of MILA and other attention mechanisms on the hybrid dataset.

Methods	ACC	Recall	Pre_w	$F1_w$	Kappa
MHRA	0.868	0.841	0.867	0.867	0.694
SHSA	0.899	0.907	0.908	0.901	0.780
Polalinear Attention	0.860	0.857	0.860	0.860	0.717
MILA	**0.907**	0.888	0.906	**0.906**	**0.786**
